# The Effect of Sexual Counseling Based on PLISSIT and EX-PLISSIT Models on Sexual Function, Satisfaction, and Quality of Life: A Systematic Review and Meta-Analysis

**DOI:** 10.1007/s10508-024-02898-2

**Published:** 2024-06-18

**Authors:** Sevil Cicek Ozdemir, Aliye Dogan Gangal, Ayten Senturk Erenel

**Affiliations:** 1 Nursing Department, Faculty of Health Sciences, Kutahya Health Sciences University, 43100 Kutahya, Turkey; 2 Nursing Department, Faculty of Health Sciences, Gazi University, Ankara, Turkey; 3 Nursing Department, Faculty of Health Sciences, Lokman Hekim University, Ankara, Turkey

**Keywords:** PLISSIT model, EX-PLISSIT model, Sexual function, Sexual satisfaction, Quality of sexual life, Meta-analysis

## Abstract

This systematic review and meta-analysis study aimed to investigate the effect of sexual counseling based on PLISSIT (Permission, Limited Information, Specific Suggestions, and Intensive Therapy) and EX-PLISSIT models on sexual function, satisfaction, and quality of sexual life. We searched seven electronic databases (MEDLINE, CINAHL, Web of Science, Cochrane Library, ProQuest, Scopus, and PubMed). Studies published between January 1, 2010, and August 16, 2022, were included in the search. Eighteen articles were eligible for inclusion in the analysis. There was a significant difference in the sexual function scores of the PLISSIT and EX-PLISSIT groups and the comparison groups (standardized mean difference (SMD): 1.677; 95% CI 0.668, 2.686; *p* < 0.05) and “sexual and communication satisfaction” sub-dimension of sexual life quality (SMD: 0.748; 95% CI 0.022, 1.475; *p* < 0.05). There was no difference in the sexual satisfaction (SMD: 0.425; 95% CI − 0.335, 1.184; *p* > 0.05) and quality of sexual life scores of the PLISSIT and EX-PLISSIT groups and the comparison groups (SMD: − 0.09; 95% CI − 0.211, 0.032; *p* > 0.05). PLISSIT and EX-PLISSIT models-based sexual counseling on sexual function was affected by the moderator variables of the time of evaluation of the results after the intervention, type of comparison group, the study population, and by whom the intervention was applied. Sexual counseling based on the PLISSIT and EX-PLISSIT models improved sexual function scores and “sexual and communication satisfaction” sub-dimension of sexual life quality.

## Introduction

Sexuality is one of the basic human needs (East & Hutchinson, 2013). Sexual function is an important part of health and one of the factors affecting the quality of life (Panahi et al., 2021). Sexual quality of life, on the other hand, refers to a general state of well-being in sexual function and satisfaction with sexual function (Mohammadi et al., 2022). In recent years, models are commonly used in sexual health assessment and counseling. It is stated that the use of models is effective in improving the sexual functions of individuals, increasing their sexual satisfaction and sexual life quality (Ziaei et al., 2022). One of these models is the PLISSIT (Permission, Limited Information, Specific Suggestions, and Intensive Therapy) and EXTENDED PLISSIT (EX-PLISSIT) models (Taylor & Davis, 2007). The PLISSIT model was first developed by Annon (1976). The model includes four levels (Permission—Limited Information—Specific Suggestions—Intensive Therapy) of intervention. Each level suggests approaches for responding to sexual concerns. The first level in meeting the sexual health needs of the individual is the evaluation process. In the second level, where information is given about the effect of the disease on sexuality and how treatment can affect sexual functions, it is emphasized that informing patients about their treatments on sexual health has an important place among nursing interventions. The third level includes special suggestions and information for the individual/partner in order to make sexual life more satisfying. The fourth level involves intensive therapy and requires referral to a specialist in sexual rehabilitation.

The PLISSIT model is a standard model and one of the most commonly used. One of the limitations of this model is its linearity and proceeding from one level to the next in which the therapist cannot diagnose the necessity of returning to the previous level to resolve the patient’s sexual concerns. Additionally, it does not include reflection and review elements. Thus, Taylor and Davis (2007) developed the EX-PLISSIT model as an extension of the PLISSIT model. EX-PLISSIT consulting model is based on the key concepts of the PLISSIT model. The main difference is that the allowing step is at the center of the other steps. As in the PLISSIT model, each step is intertwined with each other, not in sequence. In this way, it enables the individual to reveal his/her feelings and thoughts about sexuality. Counseling and intervention in the PLISSIT model, it is possible to move from one level to the other linearly, while in the EX-PLISSIT model, they are cyclical, the permission level is at the center of the other levels. Although the EX-PLISSIT model is based on the main concepts of the PLISSIT model, feedback is essential to increase self-awareness in the EX-PLISSIT model. In the EX-PLISSIT model, after seeking feedback from the client and reviewing outcomes, the therapist will be better off in challenging his or her own assumptions.

The PLISSIT and EX-PLISSIT models are used to evaluate and improve sexual health in patients with breast cancer (Keshavarz et al., 2021; Khoei et al., 2022), multiple sclerosis (Khakbazan et al., 2016), gynecological cancer (Nho, 2013), type 2 diabetes mellitus (Rutte et al., 2015), HIV positive (Asadi et al., 2018), and stoma (Ayaz, 2009). In addition, it is actively used in different processes of women's life such as pregnancy (Nejati et al., 2020) and the postpartum period (Sahin & Sentürk Erenel, 2019).

There are systematic review and meta-analysis studies on the effectiveness of the PLISSIT model in the literature (Kharaghani et al., 2020; Mashhadi et al., 2022; Tuncer & Oskay, 2022). A systematic review found that the PLISSIT model on sexual counseling was an effective, simple, useful, and cost-effective counseling method. The meta-analysis study showed that psychological interventions including the PLISSIT model improved the sexual function of women significantly (Kharaghani et al., 2020). In the other meta-analysis study, it was determined that sexual counseling based on the PLISSIT and EX-PLISSIT models was effective in sexual dysfunction (Mashhadi et al., 2022). Although there are these meta-analysis studies in the literature evaluating the effect of the PLISSIT and EX-PLISSIT models on sexual function, there are no studies in the literature examining the effect of PLISSIT and EX-PLISSIT models on sexual satisfaction and quality of sexual life. For this reason, this systematic review and meta-analysis study aimed to investigate the effect of sexual counseling based on the PLISSIT and EX-PLISSIT models on sexual function, level of satisfaction, and quality of sexual life.

## Method

### Design

It followed the Preferred Reporting Items for Systematic Reviews and Meta-Analyses (PRISMA) guideline (Moher et al., 2015). The protocol of the study was recorded in the "PROSPERO" database, which allows systematic review and meta-analysis studies to be recorded (ID: CRD42021240114).

### Search Method

We searched seven electronic databases (MEDLINE, CINAHL, Web of Science, Cochrane Library, ProQuest, Scopus, and PubMed). Studies published between January 1, 2010, and August 16, 2022, were included in the search.

The following MeSH search headings were used: “women OR female" AND plissit OR ex-plissit AND "sexual health" OR sexual OR func* OR "sexual func*” OR dysfunc* OR "sexual dysfunc*" OR "quality of life" OR satisfac* OR "sexual satisfac*" OR "sexual life." These terms and their combinations were searched as text words or abstract/title.

### Inclusion Criteria

#### Types of Studies

We limited the studies to randomized controlled trials. Randomized controlled trials and controlled trials that compared the PLISSIT and EX-PLISSIT model with control groups (e.g., usual care, placebo, no intervention, or waitlist control) or other intervention groups (BETTER model, Sexual Health Model (SHM), and solution-focused group).

#### Language of Studies

The studies published in English were included in the analysis.

#### Participants

We applied no restrictions to the participants.

#### Types of Interventions

We considered the PLISSIT and EX-PLISSIT models-based interventions. We applied no restrictions to the intervention type, dosage, duration, etc.

#### Types of Outcome Measures

The outcomes measured using validated scales were sexual function (Female Sexual Function Index [FSFI], Brief Index of Sexual Functioning for Women [BISF-W], and Sexual Dysfunctional Beliefs Questionnaire), sexual satisfaction (Hudson's Index of Sexual Satisfaction [ISI] and Berg’s Sexual Satisfaction Questionnaire), and quality of sexual life (Sexual Quality Life-Female [SQOL-F]). We imposed no restrictions for the time measuring health outcomes after the intervention (follow-up period).

### Exclusion Criteria

1. Reviews, book chapters, editorials, pilot, commentary and protocols, case repots, quasi-experimental, and duplicate articles.
2. Studies whose full text cannot be reached
3. Studies in languages other than English

### Search Outcomes

**Fig. 1:**
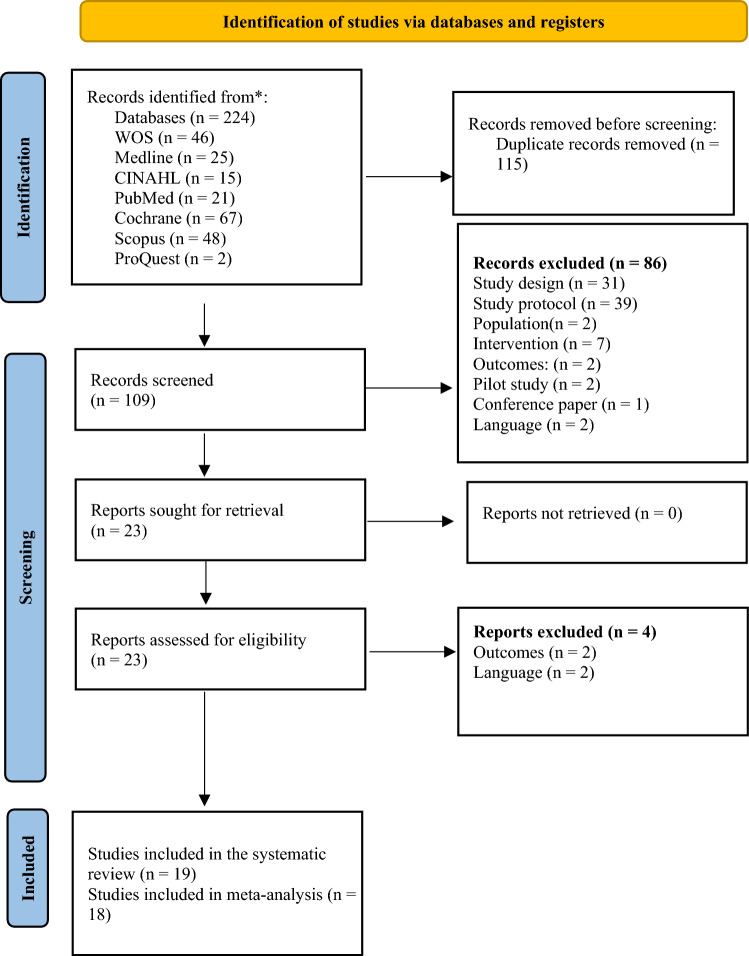
Flowchart of the literature search and study selection

We initially identified 224 records. The results were saved to a citation manager in EndNote X8-2. Then, 115 duplicated articles were identified and excluded. After removing duplicates, we excluded 86 records by reviewing titles and abstracts. The full texts of the remaining 23 records were retrieved and screened for eligibility. We excluded four articles; there were unwanted outcomes (n = 2), and they were not in English (n = 2). As a result, 19 articles were eligible for inclusion in the systematic review and 18 articles in the meta-analysis. Figure 1 presents the flowchart of this systematic review according to the Preferred Reporting Items for Systematic Reviews and Meta-Analyses (PRISMA) (Page et al., 2021). The process of study selection is illustrated in Fig. 1.

### Study Selection

Firstly, we imported the documents retrieved from seven databases into EndNote to check for duplicates. Then, the initial screening was performed by two reviewers (SCO and ADG) who independently screened the titles and abstracts of potentially relevant studies. The full texts of the studies that met the inclusion criteria were retrieved and evaluated independently for inclusion in the analysis by the two reviewers (SCO and ADG). The two researchers (SCO and ADG) read the full texts of the studies and determined whether they should be included in the analysis. If they had any disagreement during the study selection process, they tried to resolve it by trying to reach consensus. When consensus could not be reached, a third researcher (ASE) independently reviewed the full text of the study, and three researchers discussed whether it should be included in the analysis.

### Assessment of Study Quality

Two reviewers (SCO and ADG) independently assessed the study eligibility. The quality of the included studies was assessed using the RoB 2: A revised Cochrane risk of bias tool for randomized trials (Higgins et al., 2011). Before assessment, the researchers learned how to use the tool which includes seven items: (1) randomization process; (2) deviations from intended interventions; (3) missing outcome data; (4) measurement of the outcome; (5) selection of the reported result; and (6) overall bias. Each item has three options: low risk, high risk, and unclear risk. All assessments of the researchers were integrated into a risk of bias graph and risk of bias summary (Table 1).

**Table 1:** Risk of bias summary (ROB 2.0) for RCTs

	Randomization process	Deviations from intended interventions	Missing outcome data	Measurement of the outcome	Selection of the reported result	Overall bias
Asadi et al. (2018)	**?**	**–**	**–**	**?**	**?**	**–**
Azari–Barzandig et al. (2020)	**?**	**?**	** + **	**?**	** + **	**?**
Banaei et al. (2016)	**?**	**?**	**?**	**?**	** + **	**?**
Dangesaraki et al. (2019)	**?**	**?**	** + **	**?**	** + **	**?**
Farnam et al. (2014)	** + **	** + **	** + **	**?**	** + **	**?**
Ghodsi et al. (2021)	**?**	**?**	** + **	**?**	** + **	**?**
Kargar et al. (2021)	**–**	**–**	**–**	**?**	** + **	**–**
Karimi et al. (2021)	**–**	**?**	** + **	**?**	** + **	**–**
Kazemi et al. (2021)	**?**	**?**	** + **	**?**	** + **	**?**
Khakbazan et al. (2016)	**?**	**?**	**–**	**?**	** + **	**–**
Malakouti et al. (2020)	** + **	**?**	** + **	**?**	** + **	**?**
Mehrabi et al. (2019)	** + **	**?**	** + **	**?**	** + **	**?**
Moghaddam et al. (2019)	**?**	**?**	** + **	**?**	** + **	**?**
Mohammadi et al. (2022)	** + **	**–**	** + **	**?**	** + **	**–**
Nejati et al. (2020)	**?**	**–**	**?**	**?**	**?**	**–**
Rezaei–Fard et al. (2019)	** + **	**?**	**?**	**?**	** + **	**?**
Shahbazi et al. (2019)	** + **	**?**	** + **	**?**	** + **	**?**
Tutuncu and Yildiz (2012)	**?**	**?**	** + **	**?**	** + **	**?**
Ziaei et al. (2022)	** + **	**?**	** + **	**?**	** + **	**?**

+  Low risk, − High risk, and ? Some concerns

Two researchers (SCO and ADG) selected original studies on the basis of the inclusion criteria and reviewed the quality of each. Any disagreement between the two researchers regarding the assessment process was resolved through discussion. The quality assessment process was checked by the third researcher (ASE).

### Data Abstraction

Two researchers (SCO and ADG) extracted detailed data on publication information, authors’ name, publication year, publication country, publications’ name, objective, study design, participant characteristics (sample size, diagnosis, and age), intervention details, comparators, tools, outcomes, and results of each study using a structured data extraction form, which was confirmed by the third researcher (ASE).

### Data Synthesis and Analysis

All statistical analyses were performed using the Comprehensive Meta-Analysis software 3.0. All randomized studies with sufficient data to calculate the standardized mean difference were included in the meta-analyses (Higgins et al., 2022). One of the studies included in the analysis reported only median and min–max values (Dangesaraki et al., 2019). Therefore, it was not included in the meta-analysis.

Results are presented as standardized mean difference (SMD) with 95% confidence interval (CI) for continuous variables. Cochran’s Q test, *I*^2^, and Tau-squared (*τ*^2^) were performed to examine the heterogeneity. Heterogeneity was present when the Cochran’s Q test was statistically significant, and the *I*^2^ statistic was > 50% (Borenstein et al., 2011). Another indicator of heterogeneity is the Tau-squared (*τ*^2^) statistic; if the value of this test is zero, it means there is no heterogeneity (Quintana, 2015).

A random effects model was used in case of heterogeneity. We weighted the studies included in meta-analyses using the inverse-variance method. A two-sided *p* value of < 0.05 was used to indicate statistical significance. A subgroup analysis was carried out on factors that were thought to affect the homogeneity of the study. Subgroup analyses with mixed-effects models were applied to examine the potential categorical moderators for the PLISSIT or EX-PLISSIT models effects (Borenstein et al., 2011). Publication bias was assessed for the outcomes sexual function and sexual satisfaction. The funnel plot, Begg and Mazumdar’s rank correlation test, and Egger’s regression asymmetry test were used to evaluate the publication bias. There was evidence of publication bias when the results from both tests were statistically significant (Borenstein et al., 2011).

## Results

### Search Results

A total of 224 studies written in English were retrieved from all databases. Then, 115 duplicate articles were excluded. The remaining 109 articles were reviewed by title and abstract, and 86 studies that did not meet the inclusion criteria were excluded. The full texts of 23 articles were evaluated for relevance. A total of four articles whose language was not English, whose measurement outcomes were not appropriate (Fig. 1). The risk of bias of 19 articles meeting the eligibility criteria was evaluated, and they were included in the review. However, since one of these studies (Dangesaraki et al., 2019) did not report mean and SD values, it was excluded from the analysis, and 18 studies were included in the meta-analysis.

### Study Characteristics

A total of 1501 women with a mean age ranging from 18 to 60 years had participated in the 19 randomized controlled trials included in the study. One of the women constituting the sample of the evaluated studies was HIV positive, three had MS, two were post-hysterectomy, one had cyclic mastalgia, three were puerperal, one was with diabetes mellitus, two had sexual problems, three were pregnant, one high body mass index, one was gynecologic cancer, and one had spinal cord injury. Eighteen of the studies were carried out in Iran, and one was conducted in Turkey (Table 2).

**Table 2:** Data abstraction

Author/Authors Year	Country	Name of publication	Aim	Population	Age	Design	Intervention	Comparing group	Measuring tools	Measuring times	Results
Asadi et al. (2018)	Iranian	Effect of counseling based on PLISSIT model on sexual function of HIV-positive married women	To investigate the effect of counseling based on PLISSIT model on sexual function of married HIV-positive women	60 HIV-positive married women	Age ≥ 20	RCT	In the intervention group, individual counseling based on PLISSIT model was conducted in a quiet and private place by a research team member (once a week for 3 h)	Control group (routine care)	FSFI	Before intervention, 1 month, and 3 months after the intervention	The mean score of sexual functioning 1 and 3 months after the intervention showed an increase in the intervention group and a decrease in the control group. These changes in both groups were statistically significant in the intervention and in the control group
Azari-Barzandig (2020)	Iranian	The effect of counseling based on EX-PLISSIT model on sexual dysfunction and quality of sexual life of married women with multiple sclerosis (MS): A randomized controlled clinical trial	To determine the effect of counseling based on the extended PLISSIT model on sexual dysfunction and the quality of sexual life of married women with MS	70 married women with MS	18–45 age	RCT	Sexual counseling based on the EX-PLISSIT model in the intervention group (35 subjects) was conducted during a 60–90 min session by the counselor	Control group (usual care)	MSISQ-19 SQOL-F	Before interventio, 8 weeks after the intervention	The mean score of sexual dysfunction in the intervention group was significantly lower than the control group However, 8 weeks after intervention, there was a significant difference between the two groups in terms of primary and the tertiary sexual dysfunction The mean score of the quality of sexual life was not significantly different between the groups after the intervention
Banaei (2016)	Iranian	Investigating the impact of counseling based on PLISSIT model on sexual intimacy and satisfaction of breastfeeding women	To determine the impact of PLISSIT model in sexual intimacy and satisfaction of breastfeeding women referred to Health Center of Medical Science University in Bandar Abbas in 2015	100 breastfeeding women	Mean of age I: 24.93 ± 3.10 C: 23.44 ± 2.64	RCT	In the experimental group, sexual counseling was conducted based on components of PLISSIT model in two sessions of 60–90 min once per week	Control group (routine care)	Larson’s standard sexual satisfaction questionnaire	Before intervention, 1 month, and 3 months after the intervention	Three months after intervention, the mean of sexual satisfaction and sexual intimacy scores increased significantly in intervention group. There was significant difference between sexual intimacy and sexual satisfaction scores in the experimental and control groups, 1 month and 3 months after the intervention
Dangesaraki et al. (2019)	Iranian	Effect of the EX-PLISSIT model on sexual function and sexual quality of life among women after hysterectomy: A randomized controlled trial	To determine the effect of counseling based on the EX-PLISSIT counseling model on the sexual functioning and SQOL of women who underwent hysterectomy in hospitals in Sari	80 women who underwent hysterectomy	Age ≤ 50	RCT	Counseling was conducted with women in the intervention group based on the EX-PLISSIT model, in which two 1-h sessions were held on a weekly basis in the research center by a female counselor	Control group (no intervention)	FSFI SQOL-F	Before intervention, 8 weeks after the intervention	Eight weeks after the intervention, there was a significant difference in the median of FSFI scores between the intervention and control groups In addition, there was a significant difference in the median of SQOL-F scores between the intervention and control groups
Farnam et al. (2014)	Iranian	Compare the effectiveness of PLISSIT and Sexual Health Models on women's sexual problems in Tehran, Iran: A randomized controlled trial	The aim of this study was to assess whether group therapy such as Sexual Health Model (SHM) can be as effective as individual therapy like PLISSIT model in women with sexual problems	84 married women aged 20–52 years, with sexual problems	PLISSIT: 31.6 (7.4) SHM: 32.6 (7.0)	RCT	Subjects in the SHM group attended two sessions of group education, each lasting 3 h, at an interval of 1 month (up to eight women in each group). The PLISSIT group received a total of 6 h of one-on-one consultation at an interval of 1–2 weeks that were adapted from four therapeutic stages: PLISSIT model. Both programs were delivered by a trained sexual health provider (FF)	SHM	Brief Index of Sexual Functioning for Women (BISF-W)	Baseline, at 10 weeks, and at 28 weeks	Due to the considerable human resource, time, and cost spent conducting the PLISSIT, it seems that group education based on SHM could be more cost-efficient and nearly as effective
Ghodsi et al. (2021)	Iranian	The effect of sex counseling based on (permission, limited information, specific suggestions, and intensive therapy) model on sexual satisfaction in women with cyclic mastalgia: A randomized controlled clinical trial	To investigate the effect of PLISSIT model‑based sex counseling on sexual satisfaction in women with cyclic breast pain.	90 women with cyclic mastalgia	Aged from 20 years old to the menopause age	RCT	Sexual counseling based on PLISSIT as face-to-face training in four sessions (2 each week) of 90 min The intervention was performed by a counseling student in midwifery under the supervision of a tutor	Control group (Routine care)	ISS	Before intervention, 1 and 3 months following the intervention	There was a statistically significant increase in the mean score of female sexual satisfaction in the intervention group It stated that a decrease in the mean score of sexual satisfaction in the control group 1 and 3 months following the intervention
Kargar (2021)	Iranian	Comparing the Effect of Solution-Focused Group Counseling and Individual Counseling Based on PLISSIT Model on Sexual Satisfaction of Women with High Body Mass Index	This study compared the effect of solution-focused group counseling and individual counseling based on the PLISSIT model on sexual satisfaction of women with high body mass index	60 women with a BMI˃30 who	Solution-focused group counseling group: 39.30 ± 3.03 and PLISSIT-based counseling group: 39.97 ± 4.50 years	RCT	Solution-focused group counseling group received eight 45-min sessions of solution-focused counseling (twice a week). In the other group, PLISSIT-based counseling was presented in three 60-min sessions (once a week)	Solution-focused group counseling	Larson's Sexual Satisfaction Questionnaire	Before intervention, 1 month after intervention	There was a significant difference in sexual satisfaction score between two groups after intervention, and it was higher in solution-focused group counseling group (P = 0.001). Also the score of sexual satisfaction was significantly higher in the dimensions of sexual attitude (P < 0.001), quality of sex life (P < 0.001), and sexual compatibility (P < 0.001) in solution-focused group counseling group compared with individual counseling based on the PLISSIT model
Karimi et al. (2021)	Iranian	Comparing the effectiveness of sexual counseling based on PLISSIT and BETTER models on sexual self-disclosure in women with sexual problems after childbirth: A randomized trial	The aim of this study was to assess the efficacy of individual therapy using the BETTER model in comparison with individual therapy using the PLISSIT model in terms of increasing sexual self‑disclosure in women with sexual problems after childbirth	80 women with sexual problems	PLISSIT: 31.70(5.40) years BETTER: 29.70(5.50)	RCT	Women in both PLISSIT and BETTER groups attended 2 sessions of one‑on‑one consultation weekly for 2 weeks in the counseling room of the given centers. Each session lasted 60‑90 min	BETTER	FSFI	Before intervention, 1 month after intervention	The findings confirm the effectiveness of the BETTER counseling model in increasing sexual self‑disclosure after childbirth
Kazemi et al. (2021)	Iranian	The effect of counseling based on the PLISSIT model on sexual quality of life of married women with multiple sclerosis referring to MS center in 2019: A randomized, controlled trial	To investigate the effect of counseling based on the PLISSIT model on the sexual quality of life of married women with multiple sclerosis referring to MS center in Isfahan in 2019	62 women who married women with multiple sclerosis	15–49 years	RCT	The participants in the experimental group entered the study after obtaining permission from the physician and participated in four face-to-face individual training and counseling sessions, which lasted 45–75 min per session and one session per week	Control group (no intervention)	SQOL-F	Before intervention, after 2 weeks and 2 months after the intervention	Two weeks and 2 months after the intervention, the overall level of sexual quality of life in the experimental group was significantly better than the control group
Khakbazan et al. (2016)	Iranian	The effectiveness of the Permission, Limited Information, Specific suggestions, Intensive Therapy (PLISSIT) model-based sexual counseling on the sexual function of women with multiple sclerosis (MS) who are sexually active	To evaluate the effectiveness of the PLISSIT model as a valuable framework for health professionals, especially midwives to address the sexual problems of the women who are sexually active and suffer from MS	88 women who had a definite diagnosis of MS and sexual dysfunction	18–55 years	RCT	Sexual counseling was done for all women in the experimental group (EG) during 4 weeks (one 90–120 min session per week) using the one-to-one method by the same trained midwife	Control group (no intervention)	FSFI	Before the intervention, and 2 months and 3 months after the intervention	Comparison of the data collected after 2 months between the two groups showed a significant difference in the total score of FSFI. The same results were obtained when the data relating to 3 months after intervention
Malakouti et al. (2020)	Iranian	The effect of counseling based on EX-PLISSIT model on sexual function and marital satisfaction of postpartum women: A randomized controlled clinical trial	To investigate the effect of counseling based on the EX-PLISSIT model on sexual function and marital satisfaction of postpartum women	68 postpartum women	Mean of ages I: 26.6 ± 6.1 C: 27.4 ± 5.5	RCT	Sexual counseling based on the EX-PLISSIT model in a session of 60–90 min The midwife researcher participated in a training workshop on sexual problems management based on the EX-PLISSIT model taught by a sexologist	Control group (routine training)	FSFI	Before the intervention Four and 8 weeks after the end of the intervention	Mean sexual function score in the intervention group was significantly higher than the control group 8 weeks after intervention
Mehrabi et al. (2019)	Iranian	Effectiveness of sexual counseling using PLISSIT model on sexual function of women with type 2 diabetes mellitus: Results from a randomized controlled trial	To assess the effect of sexual counseling using PLISSIT model on sexual function of type 2 diabetic women	100 middle-aged diabetic women	35–55 years	RCT	At least three sessions of 45 min of individual sexual counseling were designed and developed to improve sexual function based on PLISSIT model in the intervention group by (first author)	Educational pamphlet for the control group included general care in diabetes and related therapies, nutrition, physical activity, and sexual health	FSFI	Before the first session and then 4 and 8 weeks after the intervention	Sexual function mean score had a significant change over time, and a significant difference was observed between the two groups. All subscales of sexual function in the intervention group improved except for pain and arousal
Moghaddam et al. (2019)	Iranian	Effect of counseling on the sexual satisfaction level of women with sexual dysfunction using PLISSIT model focused on dysfunctional sexual beliefs	To determine the effect of counseling on sexual satisfaction of women using the PLISSIT model focused on dysfunctional sexual beliefs	61 women with sexual dysfunction	18–49 years	RCT	One group session and three individual sessions were held based on the PLISSIT model with an interval of 1 week by midwife 1. Session 45–60 min 2. Session 90 min 3. Session 45–60 min 3. Session 45–60 min	The control group received routine care of the center, which included a general training and counseling regarding sexual health	ISS	Before intervention after intervention	Sexual counseling significantly decreased the level of sexual dysfunctional beliefs in women with sexual dysfunction, which was associated with a significant increase in the sexual satisfaction of women
Mohammadi et al. (2022)	Iranian	The effect of the EX-PLISSIT model-based psychosexual counseling on improving sexual function and sexual quality of life in gynecologic cancer survivors: A randomized controlled clinical trial	To investigate the effect of the EX-PLISSIT model-based psychosexual counseling on improving sexual function and sexual quality of life in GC survivors	110 women with the most common gynecologic (endometrial, cervical, and ovarian) cancers	18–60 years	RCT	The intervention was conducted in the form of 4 weekly group sessions 60–90 min long, held by the first author (ZM) as the intervention’s executive researcher. During each session, counseling was organized based on the EXPLISSIT framework	Control group	FSFI SQOL	At baseline and 8 weeks after the counseling sessions	The EX-PLISSIT-based psychosexual counseling resulted in positive changes in sexual function and sexual quality of life in gynecologic cancer survivors
Nejati et al. (2020)	Iranian	Effect of counseling based on the PLISSIT model on pregnant women’s sexual satisfaction: A randomized controlled trial	To evaluate the effect of PLISSIT (permission, limited information, specific suggestions, and intensive therapy) model-based sexual counseling on pregnant women’s sexual satisfaction	80 pregnant women	18–35 years	RCT	Sexual counseling based on the PLISSIT model The counseling session was conducted weekly in four sessions of 45–90 min by a trained midwife	The control group received routine consultation from the midwife of the clinic within a month,	Linda Berg’s sexual satisfaction questionnaire	Before, 2 weeks after and 4 weeks after the intervention	PLISSIT model-based sexual counseling significantly affects sexual satisfaction improvement
Rezaei-Fard et al. (2019)	Iranian	Effectiveness of sexual counseling using PLISSIT model to promote sexual function of women with spinal cord injury: A randomized controlled trial	To evaluate the effectiveness of the PLISSIT model on sexual function of women with spinal cord injury (SCI)	44 married women with SCI	18–49 years	RCT	Individuals in the intervention group received at least three 45-min sessions once a week sexual counseling using PLISSIT model by researcher (first author)	Control group (routine care)	FSFI	Before, 4 weeks after and 8 weeks after the intervention	Sexual counseling using the PLISSIT model reduced sexual problems and significantly increased the scores of sexual function, and its dimensions in women with SCI
Shahbazi et al. (2019)	Iranian	The effect of sexual counseling based on PLISSIT model on sexual function of pregnant women: A randomized controlled clinical trial	To review the effect of sexual counseling based on this model on the sexual function of pregnant women	70 pregnant women who had a sexual function	18–35 years	RCT	In the intervention group, individual and face-to-face counseling was performed in the counseling room, which was a quiet and bright room with a dimension of approximately 3*4 m and suitable for counseling. Depending on the needs of the participants, each counseling session lasted 45–60 min, held per week for consecutive weeks (1–4 sessions), and was based on the PLISSIT model	Control group (routine care)	FSFI	Before, 4 weeks after intervention	Given the effect of PLISSIT-based sexual counseling on the improvement of the sexual function of pregnant women
Tutuncu and Yildiz (2012)	Türkiye	The influence on women's sexual functions of education given according to the PLISSIT model after hysterectomy	To determine the effects of education given based on PLISSIT model to women after hysterectomy on their sexual functions	70 women who had TAH + BSO surgery	Mean of age I: 48.46 ± 6.88 C: 52.03 ± 5.41	RCT	The women within the intervention group were given individual educations ranging from 30 to 60 min, after the operation and before being discharged based on the PLISSIT model	Control group (routine clinical care)	FSFI	First and second evaluations were made during the pre-operative period. Third evaluation was made on the 3rd post-operative month; fourth evaluation was made on the 6th post-operative month by appointments	Post-operation education given to the women with TAH + BSO has positive impacts on their post-operative sexual problems and sexually-related problem solving skills
Ziaei et al. (2022)	Turkey	Comparing the Effect of Extended PLISSIT Model and Group Counseling on Sexual Function and Satisfaction of Pregnant Women: A Randomized Clinical Trial	To compare the effect of extended PLISSIT model and group counseling on Iranian pregnant women’s sexual function and satisfaction	111 pregnant women	Mean of age I:25.5 ± 4.4 C: 25.3 ± 4.9	RCT	Counseling based on EX-PLISSIT model by a trained PhD student of reproductive health under supervision of a sex therapist in Sousan Alimoradian Healthcare Center in Zanjan. Two to five sessions as needed, 30–60 min twice a week, were conducted for each pregnant woman	Control group (routine care)	FSFI ISS	Before, 4 weeks after intervention	EX-PLISSIT model and group counseling were equally effective on the sexual performance and satisfaction of pregnant women

I, Intervention group; C, Control group; RCT, Randomized controlled trial; MSISQ-19, Multiple Sclerosis Intimacy and Sexuality Questionnaire; FSFI, Female Sexual Function Index; SQOL-F, Sexual Quality of Life-Female; and ISS, Index of Sexual Satisfaction Index

The duration of the counseling session based on the PLISSIT and EX-PLISSIT was different. The number of the counseling sessions varied from 1 to 5, and the duration of each session varied from 30 to 120 min. In all studies, the comparison group was the control group, the group received routine care. The other comparison groups were the solution-focused group counseling, BETTER model, and the SHM.

The practitioners of sexual counseling based on the PLISSIT and EX-PLISSIT models had different occupations such as trained midwives (Dangesaraki et al., 2019; Khakbazan et al., 2016; Mehrabi et al., 2019; Moghaddam et al., 2019; Nejati et al., 2020), research team members (Asadi et al., 2020; Farnam et al., 2014; Kargar et al., 2021; Malakouti et al., 2020; Mohammadi et al., 2022; Rezaei-Fard et al., 2019; Tutuncu and Yilmaz, 2012), a counselor (Azari-Barzandig et al., 2020), and a trained student of reproductive health under the supervision of a sex therapist or tutor (Ghodsi et al., 2021; Ziaei et al., 2022).

Ten studies used the FSFI to evaluate sexual function (Asadi et al., 2018; Dangesaraki et al., 2019; Karimi et al., 2021; Khakbazan et al., 2016; Malakouti et al., 2020; Mehrabi et al., 2019; Mohammadi et al., 2022; Rezaei-Fard et al., 2019; Shahbazi et al., 2019; Tutuncu & Yıldız, 2012), and one study used the Brief Index of Sexual Functioning for Women (BISF-W) (Farnam et al., 2014). The measurement times differed in these studies. In some studies, the FSFI was administered 1 month after the intervention (Asadi et al., 2018; Karimi et al., 2021; Malakouti et al., 2020; Mehrabi et al., 2019; Rezaei-Fard et al., 2019; Shahbazi et al., 2019), while in some others, it was administered 2 months after the intervention (Khakbazan et al., 2016; Malakouti et al., 2020; Mehrabi et al., 2019; Mohammadi et al., 2022; Rezaei-Fard et al., 2019). It was administered 3 months after the intervention in three studies (Asadi et al., 2018; Khakbazan et al., 2016; Tutuncu & Yıldız, 2012). In one study, sexual function was evaluated by administering the FSFI 6 months after the intervention (Tutuncu & Yıldız, 2012), and again only one study used the Sexual Dysfunctional Beliefs Questionnaire (Moghaddam et al., 2019). And also in one study, sexual function was evaluated by administering the BISF-W 7 months after the intervention (Farnam et al., 2014).

In five studies, sexual satisfaction was evaluated using the ISS (Banei et al., 2016; Ghodsi et al., 2021; Kargar et al., 2021; Moghaddam et al., 2019; Ziaei et al., 2022). One study used Linda Berg's Sexual Satisfaction Questionnaire to assess sexual satisfaction (Nejati et al., 2020). In almost all studies, sexual satisfaction was evaluated 1 month after the intervention (Banei et al., 2016; Ghodsi et al., 2021; Kargar et al., 2021; Moghaddam et al., 2019; Nejati et al., 2020; Ziaei et al., 2022). One study evaluated sexual satisfaction 2 weeks after the intervention (Nejati et al., 2020), and two studies evaluated it 3 months after the intervention (Banei et al., 2016; Ghodsi et al., 2021).

Sexual quality of life was evaluated in four studies. The SQOL-F was used as a measurement tool for this purpose (Azari-Barzandig et al., 2020; Dangesaraki et al., 2019; Kazemi et al., 2021; Mohammadi et al., 2022). In these studies, sexual quality of life was reevaluated 2 months after the intervention. It was evaluated 2 weeks after the intervention in only one study (Kazemi et al., 2021).

### Risk of Bias

The bias assessment of nineteen RCT studies using the Cochrane risk of bias ROB 2.0 instrument showed that six studies were at high risk, and thirteen studies were at some concerns. These studies lacked detailed randomization methods and allocation concealment. And also data collection was based on self-report questionnaires, the participants or practitioners could not be blinded and no “intention-to-treat estimates.” Table 1 shows for the details summarizing the risk of bias assessment for the included studies.

### Effectiveness of the PLISSIT and EX-PLISSIT Models

#### Sexual Function

**Fig. 2:**
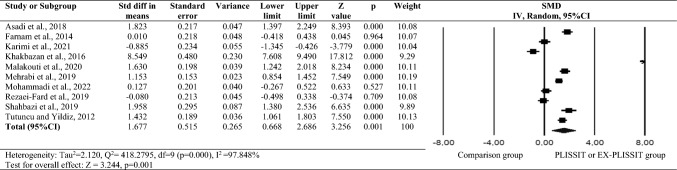
Meta-analyses for sexual function. SMD: standardized mean difference; CI: confidence interval; IV: inverse variance

Outcome data 1, 2, 3, 6, or 7 months after the intervention were available in nine trials (1121 women). Random effects was selected, because the ten studies were heterogeneous (Tau^2^ = 2.120, *Q*^2^ = 418.2795, d*f* = 9 (*p* < .001), *I*^2^ = 97.848%). The forest plot in Fig. 2 illustrates that there was a significant difference in the sexual function scores of the PLISSIT and EX-PLISSIT groups and the comparison group (SMD: 1.677; 95% CI 0.668, 2.686; *p* < 0.05).

#### Sexual Satisfaction

**Fig. 3:**
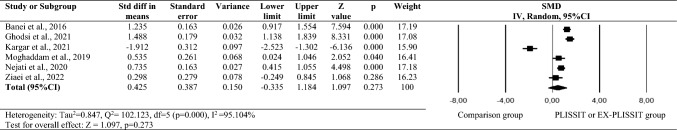
Meta-analyses for sexual satisfaction. SMD: standardized mean difference; CI: confidence interval; IV: inverse variance

Outcome data were available for six trials (673 women). Random effects was selected because these six studies were heterogeneous (Tau^2^ = 0.847, *Q*^2^ = 102.123, d*f* = 5 (*p* < .001), *I*^2^ = 95.104%). The forest plot in Fig. 3 illustrates that there was not significant difference in the sexual satisfaction scores of the PLISSIT and EX-PLISSIT groups and the comparison group (SMD: 0.425; 95% CI − 0.335, 1.184; *p* > 0.05).

#### Quality of Sexual Life

**Fig. 4:**
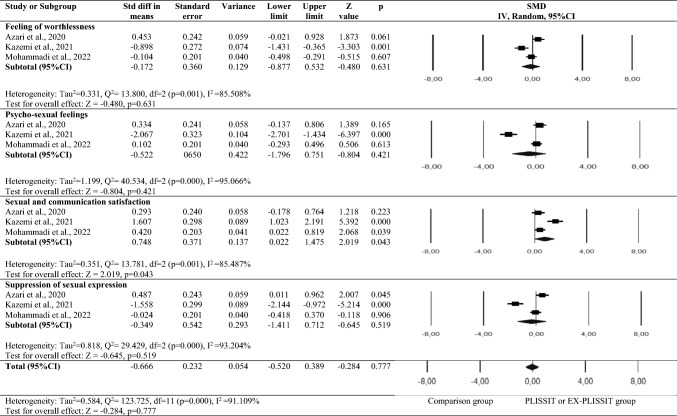
Meta-analyses for quality of sexual life. SMD: standardized mean difference; CI: confidence interval; IV: inverse variance

Outcome data were available for three trials (1164 women). Random effects was selected because these three studies were heterogeneous (Tau^2^ = 0.584, *Q*^2^ = 123.725, d*f* = 11 (*p* < .001), *I*^2^ = 91.109%). The forest plot in Fig. 4 illustrates that there was no difference in the quality of sexual life scores of the PLISSIT and EX-PLISSIT groups and the comparison group (SMD: − 0.666; 95% CI − 0.520, 0.389; *p* > 0.05). SQOL-F scale sub-dimension scores of the groups were examined. No difference was found between the PLISSIT and EX-PLISSIT groups and the comparison group in terms of the mean “feeling of worthlessness” (SMD: − 0.172; CI − 0.877, 0.532; *p* > 0.05), “psycho-sexual feelings” (SMD: -0.522, CI − 1.796, 0.751; *p* > 0.05), and “suppression of sexual expression” (SMD: − 0.349, CI − 1.411, 0.712; *p* > 0.05) sub-dimension scores of the SQOL. However, a significant difference was observed between the PLISSIT and EX-PLISSIT groups and the comparison group in terms of the mean “sexual and communication satisfaction” sub-dimension score (SMD: 0.748; 95% CI 0.022, 1.475; *p* < 0.05).

#### Subgroup Analysis

Two months (5.588) and 3 months (4.170) after intervention had a greater effect in improving sexual function than those with other measurement times (effect size: 2.489; 95% CI 1.256, 3.721; *p* < 0.05). In studies with a control group, the effect of the PLISSIT and EX-PLISSIT models on sexual function is higher than other studies (BETTER and SHM groups) (effect size: 1.514; 95% CI 0.614, 2.414; *p* < 0.05). The effect of PLISSIT and EX-PLISSIT models on sexual function of women with diseases (2.792) and with hysterectomy (1.432) was more than in women with pregnant or postpartum (0.899), with cancer (0.127), and with sexual problems (effect size: 1.539; 95% CI 0.327, 2.751; *p* < 0.05). The intervention is performed by a trained midwife (4.699) which had a greater effect in improving sexual function than by a research team members (0.824) (effect size: 1.770; 95% CI 0.685, 2.854; *p* < 0.05) (Table 3).

**Table 3:** Results of subgroups analysis

Categorical moderators	Sexual function					Sexual satisfaction				
	*k*	Effect size	95%CI	*p* value	*Q* between df *p*	*k*	Effect size	95%CI	*p* value	*Q* between d*f* *p*
*Time of the outcome evaluation*										
One month after intervention	6	0.181	− 1.771, 2.133	.856	14.732 d*f* = 4 0.005	6	0.309	− 0.475, 1.094	0.440	19.839 d*f* = 2 0.000
Two months after intervention	5	5.588	3.230, 7.946	.000		1	0.569	0.122, 1.016	.013	
Three months after intervention	3	4.170	1.379, 6.962	.003		2	2.042	1.482, 2.603	.000	
Six months after intervention	1	1.155	− 3.622, 5.932	.636						
Seven months after intervention	1	0.010	− 4.760, 4.779	.997						
Overall	16	2.489	1.256, 3.721	.000		9	1.004	0.685, 1.323	.000	
*Type of comparison group*										
BETTER group	1	− 0.885	− 3.726, 1.955	.541	4.736 d*f* = 2 0.094					
SHM group	1	0.010	− 2.826, 2.846	.995						
Control group	8	2.006	0.998, 3.013	.000		5	0.892	0.485, 1.299	.000	56.033 d*f* = 1 0.000
Solution-focused group						1	− 1.912	− 2.523, − 1.302	.000	
Overall	10	1.514	0.614, 2.414	.001		6	0.029	− 0.310, 0.368	.869	
*The study population*										
Women with sexual problems	1	0.010	− 3.816, 3.835	.996	3.096 4 0.542	1	0.535	− 1.830, 2.899	0.658	0.739 2 0.691
Women with hysterectomy	1	1.432	− 2.387, 5.252	.462						
Women with cancer	1	0.127	− 3.695, 3.949	.948						
Pregnant or postpartum women	3	0.899	− 1.314, 3.111	.426		3	0.761	− 0.592, 2.115	.270	
Women with diseases (MS, HIV, SCI, diabetic, CM, and HBMI)	4	2.792	0.870, 4.714	.004		2	− 0.174	− 1.843, 1.496	.838	
Overall	10	1.539	0.327, 2.751	.013		6	0.414	− 0.546, 1,375	.398	
*By whom the intervention*										
Trained midwives	2	4.699	2.503, 6.895	.000	9.039 1 0.003	2	0.641	− 0.160, 1.442	.117	15.757 2 0.000
Research team members	6	0.824	− 0.423, 2.072	.195		1	− 1.912	− 3.129, − 0.696	.002	
Trained student						2	0.933	0.123, 1.743	.024	
Overall	8	1.770	0.685, 2.854	.001		5	0.3	− 0.215, 0.816	.253	

*k*, number of samples; SHM, Sexual Health Model; MS, multiple sclerosis; SCI, spinal cord injury; CM, cyclic mastalgia; and HBMI, high body mass index

Three months of after intervention (2.042) had a greater effect in improving sexual satisfaction than those with 1 and 2 months (1.004, 95% CI 0.685, 1.323, *p* < 0.05). There was not significant overall effect of PLISSIT and EX-PLISSIT models on sexual satisfaction by the comparison group, study population, and by whom intervention (*p* > 0.05) (Table 3).

#### Publication Bias

**Fig. 5:**
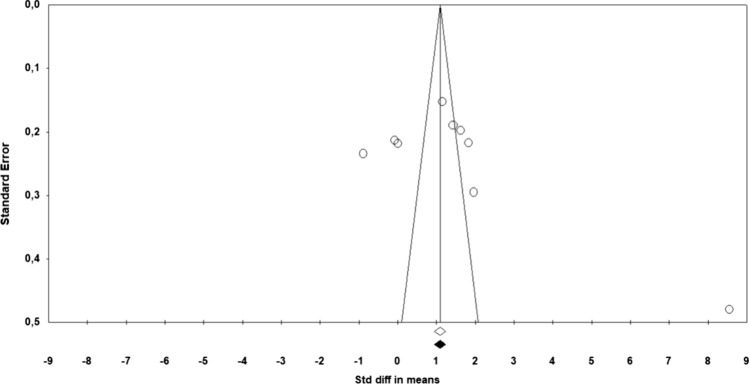
Funnel plot for sexual function

**Fig. 6:**
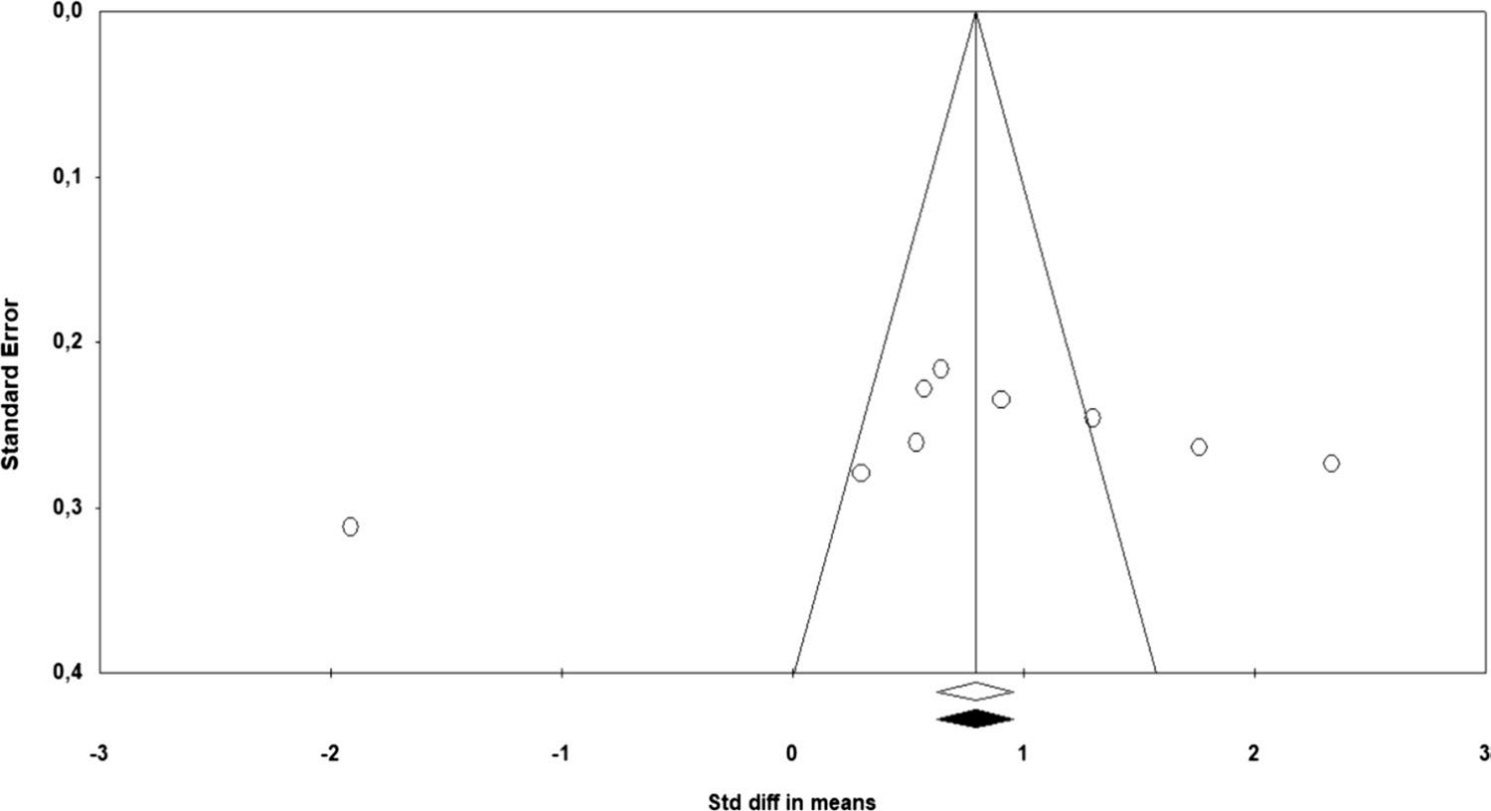
Funnel plot for sexual satisfaction

Based on the results obtained using the funnel plot of standard error by SMD for the outcomes sexual function and sexual satisfaction, it was not possible to conclude that there was a likelihood of publication bias (Figs. 5 and 6). Because the Begg and Mazumdar rank correlation test (*p* = 0.465) and the Egger’s regression test (*p* = 0.138) were not significant for the outcomes sexual function. Likewise, the Begg and Mazumdar rank correlation test (*p* = 0.916) and the Egger’s regression test (*p* = 0.408) were not significant for the outcomes sexual satisfaction.

## Discussion

Our meta-analysis revealed that individual sexual counseling based on the PLISSIT and EX-PLISSIT models applied in different populations improves sexual. And also, there was a positive and significant improvement only in the “sexual and communication satisfaction” sub-dimension of sexual life quality.

Sexuality is affected by many physiological, cultural, social, and psychological factors (McCool-Myers et al., 2018). This effect is more pronounced in pathologies that are directly related to reproduction rates, and in medical conditions such as multiple sclerosis and spinal cord injury that affect the muscle and neurological system (Azimi et al., 2019; Courtois et al., 2017). In addition, it is known that chronic diseases and periods such as pregnancy, postpartum period, and menopause, where biological and hormonal changes are experienced, directly affect sexual life negatively (Sentürk Erenel et al., 2015; Gutzeit et al., 2020; Rahmanian et al., 2019). The women in our study had different characteristics (HIV positive, MS, women with hysterectomy, pregnant women, postpartum women, cancer, with diabetes mellitus, women with sexual problems, high body mass index, and spinal cord injury). The study revealed that individual sexual counseling based on the PLISSIT and EX-PLISSIT models had a positive effect on sexual function. Similar to our results, two systematic review studies which examined the effectiveness of sexual counseling based on the PLISSIT model reported that the use of a model was effective in improving sexual functions (Kırıcı & Ege, 2021; Tuncer & Oskay, 2022). One meta-analysis study which investigated the effect of sexual counseling based on the PLISSIT model on sexual dysfunction in both women and men reported that counseling positively affects sexual function (Mashhadi et al., 2022).

Our meta-analysis showed that individual sexual counseling based on the PLISSIT and EX-PLISSIT models had a positive effect on the sub-dimension of sexual and communication satisfaction in the SQOL scale. It is known that with the increase in sexual function, the harmony between the couples increases and the general quality of life is positively affected (Jones et al., 2018; Mallory et al., 2019). In this context, our meta-analysis findings support the literature. However, no significant effect was observed in this study in the other sub-dimensions of the SQOL scale (feeling of worthlessness, psychosexual feelings, and suppression of sexual expression). This may be related to the multidimensional and complex nature of sexuality.

According to the subgroup analysis, it was determined that the effectiveness of sexual counseling based on PLISSIT and EX-PLISSIT model on sexual function and satisfaction was high in the 2nd and 3rd months. However, the effect of PLISSIT and EX-PLISSIT model on sexual function decreased in the 6th and 7th months. This shows that the effectiveness of the counseling based on the PLISSIT and EX-PLISSIT models decreased in the following periods. It is important to evaluate the effects of sexual counseling at certain periods and to repeat the intervention when necessary (Kharaghani et al., 2020).

In studies with a control group, the effect of the PLISSIT and EX-PLISSIT models on sexual function is higher than other studies (BETTER and SHM groups). This may have resulted from the fact that the individuals participating in the control group studies did not receive any intervention and received routine care.

There was no standardized model-based counseling in the reviewed studies, and counseling was carried out by different practitioners with different populations. According to the subgroup analysis, the effectiveness of sexual counseling based on PLISSIT and EX-PLISSIT model on sexual function is higher in women with diseases (MS, HIV, SCI, diabetes mellitus, CM, and obesity) and post-hysterectomy, than in women with sexual problems and pregnant or postpartum women. Sexual interest and desire decrease due to physical and physiological changes during pregnancy and postpartum. The frequency of sexual intercourse during pregnancy decreases due to cultural practices, lack of awareness that sexual intercourse during pregnancy is not contraindicated unless recommended by an obstetrician, belief that it may cause miscarriage, stillbirth and fetal infections, lack of appropriate counseling by health-care providers about safe sexual practices during pregnancy, and lack of communication between spouses about their sexual expectations and needs during pregnancy (Fernández-Carrasco et al., 2020; Thapa et al., 2023). During the puerperium, sexual interest and desire decreases due to body changes, pain, fatigue, anxiety, and role changes (Drozdowskyj et al., 2020). It is thought that the reason why the effectiveness of the models is less effective in women during pregnancy and puerperium is due to the decrease in sexual interest and desire in women during these processes and the focus being on the baby.

Furthermore, according to subgroup analysis, we identified that the trained midwifery by sexual counseling based on PLISSIT and EX-PLISSIT models was an important moderator on increasing sexual function. This may be due to the longer duration of counseling provided by trained midwives. In addition, midwives, like nurses, are the main care providers who first contact with the patient and are perceived as more reliable by patients (Demir et al., 2020). These situations may have affected the high effectiveness of midwife-led interventions.

Studies in this meta-analysis were rated to have “some concerns” or “a high risk of bias.” The reviewed studies did not include blinding, and the data were collected using self-report questionnaires, which increased the risk of bias. In addition, "allocation concealment" was not ensured during the allocation process, and no appropriate analyses were used in the evaluation of the missing data, which increased the risk of bias in the reviewed studies. Therefore, it is recommended to evaluate the results of this meta-analysis considering the risk of bias findings. Experimental studies with low risk of bias are needed to clearly demonstrate the effect of individual counseling based on the PLISSIT and EX-PLISSIT models on sexual health parameters.

### Conclusion and Recommendations

This meta-analysis showed that sexual counseling based on the PLISSIT and EX-PLISSIT models provided significant improvements in sexual function and “sexual and communication satisfaction” sub-dimension of sexual life quality. According to the subgroup analysis, it was determined that PLISSIT and EX-PLISSIT models-based sexual counseling on sexual function was affected by the moderator variables of the time of evaluation of the results after the intervention, type of comparison group, the study population, and by whom the intervention was applied. In addition, there is a need for more methodologically strong experimental studies in this area in order to explain the current effect more clearly.
